# Tuberculous Spondylodiscitis: A Report of Two Cases and Literature Review

**DOI:** 10.7759/cureus.85235

**Published:** 2025-06-02

**Authors:** Ivo I Kehayov, Atanas N Davarski, Georgi S Slavov, Borislav D Kitov

**Affiliations:** 1 Department of Neurosurgery, Faculty of Medicine, Medical University of Plovdiv, Plovdiv, BGR; 2 Department of Neurology, Faculty of Medicine, Medical University of Plovdiv, Plovdiv, BGR

**Keywords:** magnetic resonance (mr), paravertebral abscess, pott’s disease, treatment choices, tuberculous spondylitis

## Abstract

Tuberculosis ranks as one of the most deadly infectious diseases globally. While it primarily attacks the lungs, it can also develop in other parts of the body. It can involve the vertebral column, a condition known as Pott’s disease. How often it occurs, what symptoms appear, and how severe it becomes all depend on which spinal segments are involved and how much bone has been damaged. Because the signs of Pott’s disease are varied and often vague, even modern healthcare systems can struggle to diagnose it promptly. This delay is the main reason why many patients go on to suffer neurological impairments, spinal instability, and generally poor outcomes, often without ever regaining full neurological function. In this report, we describe two cases of tuberculous spondylodiscitis, highlighting their clinical presentations and imaging results, and we review the literature to outline the most effective strategies for diagnosis and treatment.

## Introduction

Tuberculosis is the second leading cause of death from infectious diseases worldwide [1]. In 2018, an estimated 10 million people were infected with tuberculosis, of whom 1.5 million died [2]. It most commonly affects the lungs, but it can also occur outside the pulmonary system, resulting in extrapulmonary tuberculosis. Approximately 5% of the cases of extrapulmonary tuberculosis are infections of the vertebral bodies, called Pott's disease [3].

The clinical presentation of Pott's disease is diverse and nonspecific, making timely diagnosis a significant challenge in both developed and developing countries [4]. Due to the insidious nature of the disease, patients often do not develop neurological deficits until severe spinal deformities with spinal cord compression have developed. According to Colmenero et al., the time from initial symptom onset to diagnosis ranges from four to six months [5]. Delay in diagnosis is the most important factor contributing to the development of neurological deficits, segmental instability, and poor prognosis, with a significant number of patients never regaining neurological function [4]. Magnetic resonance imaging (MRI) is able to identify soft tissue changes in spinal tuberculosis, but is usually used in the presence of neurological symptoms and is mistaken for nontuberculous spinal infection [4].

Treatment of early-stage Pott's disease with standard antituberculosis regimens results in a cure rate of approximately 95% of patients, demonstrating the crucial importance of early diagnosis [6]. Unfortunately, Pott's disease is rarely suspected by physicians, especially those in low-incidence countries [5]. On the other hand, patients often present with nonspecific symptoms and misleading findings, such as a negative skin test for purified protein derivatives, despite the presence of active infection [4]. To improve the timely diagnosis of tuberculous spondylodiscitis (TS), it is necessary to increase awareness of both the various clinical manifestations and the specific diagnostic features of the disease.

The purpose of this article is to present two cases of TS, with an emphasis on diagnosis and treatment according to literature data, especially in Bulgaria, to improve awareness among specialist neurologists and neurosurgeons, which will serve as a prerequisite for faster diagnosis and adequate treatment.

## Case presentation

Case 1

A 70-year-old female patient had been complaining of neck and upper back pain for six months. Her walking gradually became more and more difficult. Three months later, a computed tomography (CT) scan showed evidence of spondylodiscitis at the T1-T2 level, which is why a decompressive laminectomy was performed at that level in another hospital. In the following months, the pain in the neck and thoracic region intensified and spread to the right arm, and the legs became almost immobilized, making walking impossible. 

**Figure 1:**
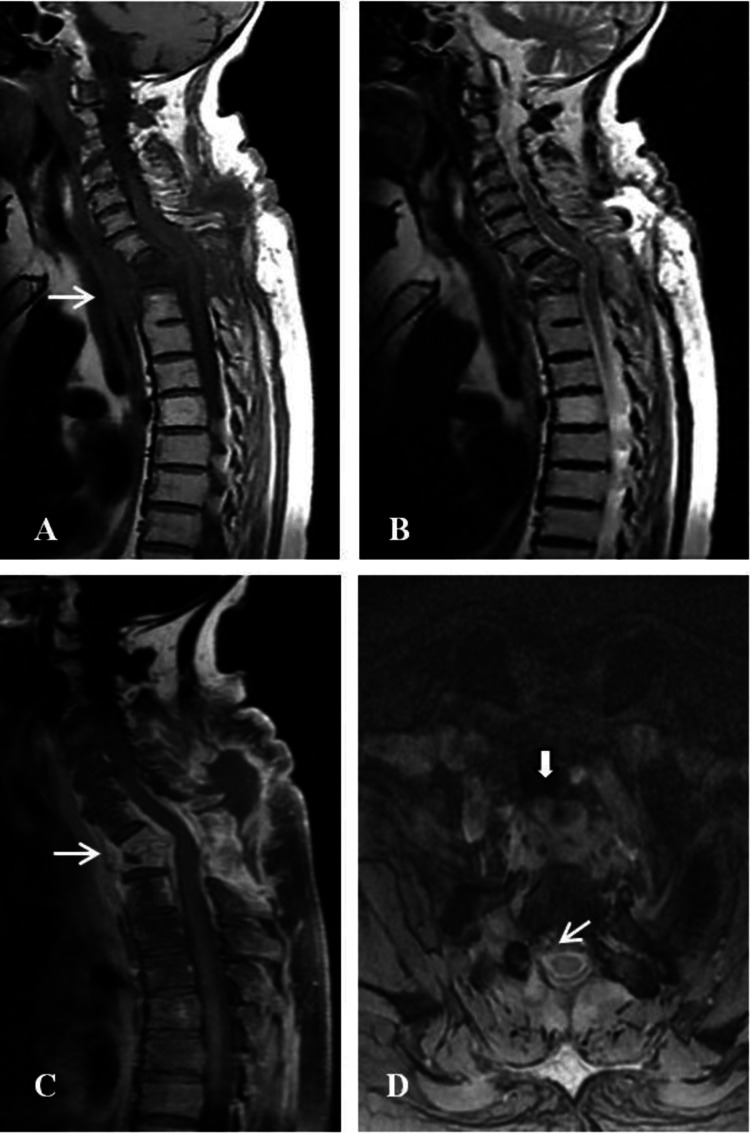
MRI of the patient with tuberculous spondylodiscitis at the T1 – T2 level (Case 1) (A) Sagittal T1 projection – hypointense signal with subligamentous spread and presence of paravertebral abscess (thin solid arrow); (B) Sagittal T2 projection – inhomogeneous hyperintense signal; (C) Sagittal T1 projection with contrast – focal inhomogeneity and enhancement of certain intraosseous structures and paravertebral abscess (thin solid arrow); (D) Axial T1 projection with contrast – well-defined ventral paravertebral abscess (thick solid arrow) and epidural abscess (thin solid arrow).

On this occasion, she was admitted to the Neurosurgery Clinic. The examination revealed painful kyphosis at the T1-T2 level, severe lower paraparesis (possible minimal movements in supine position 1/5 symmetrical), vivid tendon-periosteal reflexes, Babinski ++ bilaterally, distal conductive hypesthesia from T4, impaired deep sensation, and retention of the pelvic reservoirs. Blood tests show elevated erythrocyte sedimentation rate (ESR) and C-reactive protein (CRP), but a normal white blood cell count (Table 1). The MRI performed revealed pronounced spondylodiscitis at the T1-T2 level, which was defined as tuberculous based on the destruction of the affected vertebral bodies > 50%, subligamentous spread cranially and distally, and the presence of paravertebral, intervertebral, and epidural abscesses. The T-spot test performed was positive for active tuberculosis disease (Figure 1).

**Table 1:** Laboratory blood test (Case 1)

Indicator	Patient Value	Reference Range
Leukocytes (WBC)	7.4 х 10^9^/gl.	3.5-10.5 х 10^9^ g/l
Erythrocyte Sedimentation Rate	60 mm/h	0-20 mm/h
C-reactive Protein	37 mg/L	≤ 8 mg/l

**Figure 2:**
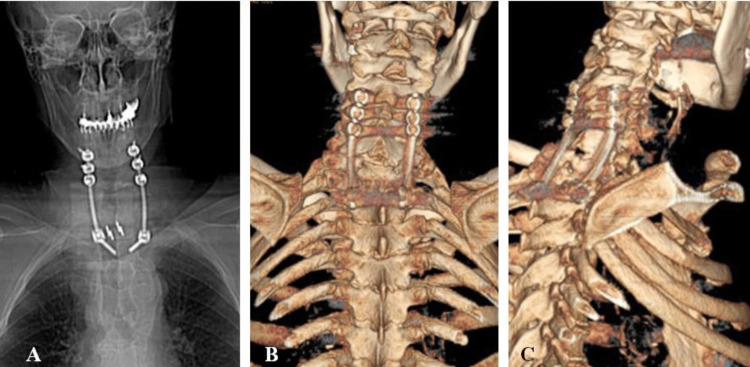
Postoperative crevicothoracic CT scan and 3D reconstruction (Case 1) (A) posterior long segment stabilization and implanted cage T1-T2; (B, C) Postoperative CT 3D reconstruction (front and profile) – good reconstruction and stabilization 3D: three-dimensional

Given the picture and imaging findings, the patient was reoperated. The old surgical scar expanded proximally and caudally, centered at the T1 level. The bilateral disinsertion of the paravertebral muscles on the right extended along the course of the first and second ribs. The previously performed laminectomy was extended, and right-sided costotransversectomy was also performed at the T1 and T2 levels, resecting the transverse processes, pedicles, and the first and second ribs in their proximal sections to the costotransverse joints. The access provided allowed for a discectomy at the T1-T2 level, in which inflammatory material from the disc with necrosis and decay, and a certain amount of liquid pus was removed. The existing epidural abscess was evacuated. A posterolateral fusion with a titanium-carbon cage was performed in the area of the T1-T2 disc space. Posterior stabilization was also performed with the implantation of six screws in the lateral mass at the C4, C5 and C6 levels and two transpedicular screws at the T3 level, which were connected with two titanium rods, which allowed for good alignment of the cervicothoracic junction (Figure 2).

Postoperatively, regression of the lower paraparesis (2/5 symmetrical) was noted to the extent of independent walking with a walker. The patient was referred to a rehabilitation ward for continued treatment. At the follow-up examination a month later, the patient was moving independently with a cane (3/5 symmetrical); superficial sensation and function of the pelvic reservoirs were restored.

Case 2 

A 36-year-old man with a history of incision of swollen lymph nodes at the base of the neck, as well as a family member suffering from tuberculosis. For about five to six months, he had been complaining of a gradual curvature of the spine and a progressive weakening of the strength (2/5 symmetrical) and sensitivity of the lower extremities at level T10. Two months before admission to the clinic, after a fall, the lower back pain had significantly increased. Gradually, his walking became very difficult, and his spine significantly curved to the left. After a consultation and imaging tests, the patient was admitted to the Neurosurgery Clinic for treatment.

**Figure 3:**
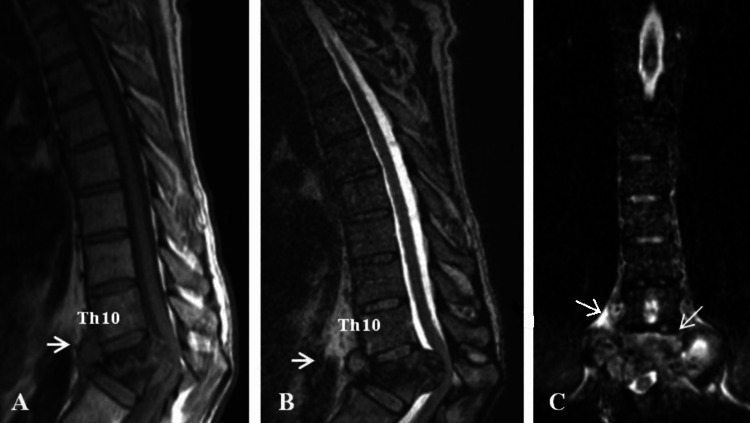
MRI of the tuberculous spondylodiscitis at the level of T11 – T12 (Case 2) (A) Sagittal projection T1 – heterogeneous hypointense signal with pronounced bone marrow edema, subligamentous spread of the infection, with pronounced compression of the spinal cord and paravertebral abscess (thin solid arrow); (B) Sagittal projection T2 shows heterogeneous hyperintense signal, compression of the spinal cord and paravertebral abscess (thin solid arrow); (C) Coronal projection T2 stir visualizes well the paravertebral abscess around the affected segments (thin solid arrows).

Upon admission to the clinic, a gross vertebral syndrome in the lower thoracic and lumbar region and kyphoscoliosis with a maximum punctum at T12 were detected. The neurological status showed a lower flaccid paraparesis (2/5 symmetrical), conductive hypoesthesia distal to the umbilicus, and partial retention of the pelvic reservoirs at level T10. The blood test showed mildly elevated leukocytes and CRP and a markedly elevated ESR (Table 2). The performed MRI of the spine visualized a pronounced wedge-shaped deformity with severe inflammatory changes at the level of T11-T12 vertebrae, with significant > 50% destruction of their anterior columns, leading to protrusion of infected sequesters into the vertebral canal with medullary compression and myelopathic focus. There was pronounced cortical bone marrow oedema and gross hypointense obliteration of the endplate and body of T12 vertebra and severe osteolytic osteomyelitis with reduction of the bone structure of the vertebral bodies, partial nucleolysis of the T11-T12 and T12-L1 discs (Figure 3). The MRI findings were interpreted as TS. The performed tuberculosis spot test was positive for active tuberculosis disease, and pulmonography showed an infiltrative pneumonic form of tuberculosis, in the decay phase.

**Table 2:** Laboratory blood test (Case 2)

Indicator	Patient Value	Reference Range
Leukocytes (WBC)	13.1 х 10^9^ g/l	3.5-10.5 х 10^9^ g/l
Erythrocyte Sedimentation Rate	79 mm/h	0-20 mm/h
C-reactive Protein	14.3 mg/l	≤ 8 mg/l

**Figure 4:**
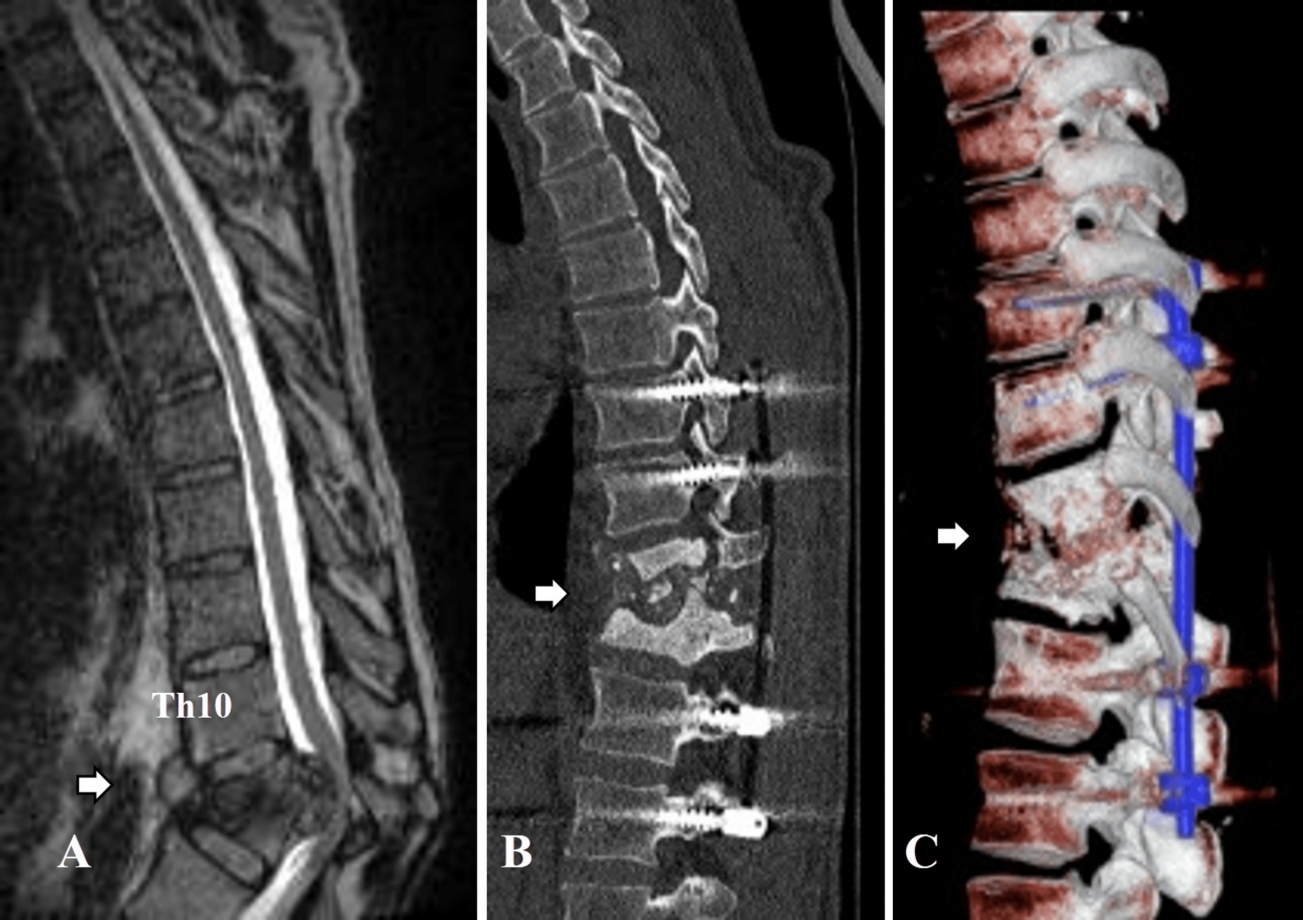
Preoperative and postoperative imaging (Case 2) (A) Preoperative MRI T2 (thick solid arrow); (B) Sagittal postoperative CT reconstruction – shows the achieved alienation of the affected vertebrae and the instrumentation performed (thick solid arrow); (C) Three-dimensional reconstruction (thick solid arrow).

Given the pronounced neurological deficit and MRI findings, the patient underwent surgery. A wide decompressive laminectomy of T11 and T12 was performed with extraction of the medial and lateral articular facets. Epidural pathological inflammatory granulomatous tissue and bone sequestra compressing the spinal cord were found, which were excised. Satisfactory decompression of the medulla spinalis was performed, as well as aseptic lavage and drainage. Considering the pronounced pathological mobility, dorsal transpedicular instrumentation was performed with 8 6.5/50 titanium screws CD Horizon™ Legacy® Spinal System (Medtronic plc, Galway, Ireland), titanium alloy, polyaxial fixed angle screws at the level of T9 - T10 - L1 - L2. Two pre-modelled and sized metal rods (titanium alloy - 180) were implanted bilaterally, which allowed definitive stabilization with adequate physiological alignment of the vertebral bodies (Figure 4). The histological result showed the presence of hyalinized connective tissue, an abundance of calcifications, focal round cell inflammatory infiltration, and detritus.

Postoperatively, the patient's condition gradually improved; the vertebral syndrome regressed in the following days, and the lower back pain decreased. Kyphoscoliosis was reduced to a certain extent. Lower paraparesis regressed to 4/5 symmetrical, and the pelvic reservoir functions were restored.

## Discussion

The overall incidence of spinal infections in adults is approximately 2.2 per 100,000 per year, with TS accounting for 17-40% of all cases of infectious spondylodiscitis [7].

The spine is the most common location of musculoskeletal tuberculosis, accounting for 1-2% of all tuberculosis cases [8]. According to the National Program for Prevention and Control of Tuberculosis in the Republic of Bulgaria for the period 2021-2025, the incidence of tuberculosis in the country in 2019 was 18.5 cases per 100,000 people, which approximately translates to 0.18-0.37 cases of TS per 100,000 people [9].

There is no definitive data in the literature on the gender distribution of patients with TS. In some studies, the female sex predominates from 0.65:1 to 0.85:1 [10,11]. In the study by Shetty et al., which included 66 patients, the male-to-female ratio was 1.2:1, and the systematic literature review by Ferrer et al., based on 37 publications, found a ratio of 1.1:1 [12,13]. In the current report, we had one female patient and one male patient.

The literature generally suggests that TS affects younger patients, 45-60 years of age [14]. Ferrer et al. reported a mean age of 43.4 years (range 9-76 years), which is consistent with the mean age of 49.04 ± 13.70 years found by Vulpe et al., which is statistically lower than that of patients with pyogenic spondylodiscitis [10,13]. According to Colmenero et al., two peaks in the occurrence of TS are observed: between 20 and 30 years, associated with immigration and HIV, and between 60 and 70 years, associated with immunosuppression and comorbidities of the patients [15]. This is also confirmed by Shetty et al., who described 66 cases of patients with TS aged ≥ 60 years [12]. The ages of our two patients were 46 and 70 years, which is consistent with the data reported in the literature and indicates that age is not an important diagnostic factor.

In TS, the symptoms are nonspecific and develop insidiously, due to the slow progression of the disease, which contributes to a significant prolongation of the period from its occurrence to diagnosis, which varies even in the most developed countries between three and six months [7,11,16]. The two cases in the current report are similar to this.

The disease usually presents with spinal pain (83-100% of patients), which may be moderate to severe, axial or radicular, and often localized in the affected area [16]. The severity of the pain is directly related to the degree of vertebral destruction, the development of pathological fractures, and nerve root compression [17]. Spinal deformity and the development of neurological deficits are the worst complications of TS. Despite the progress in diagnostic techniques in the developed countries, neurological deficits are present at diagnosis in ≥ 49.7% of cases, as confirmed by the clinical presentation of our patients [7]. Neurological deficits result from mechanical compression of the spinal cord by abscess, granulation tissue, or sequestration, or pathological instability. In rare cases, sudden neurological deficits may occur as a result of vascular compression by bone sequestration or inflammatory vascular thrombosis of the vertebral arteries. Once neurological deficit develops, a significant number of patients may never regain their neurological function [4]. Treatment of early-stage Pott's disease with standard treatment regimens results in a cure in approximately 95% of patients, clearly demonstrating that early diagnosis is crucial in preventing the development of neurological deficits [6]. Because of the low incidence of the disease and nonspecific clinical symptoms, physicians often have a low index of suspicion for TS.

Laboratory tests

Inflammatory markers such as ESR, WBC count, neutrophil percentage, and CRP level are highly sensitive in the diagnosis of bone and joint infection, but are not specific [11]. According to Lertudomphonwanit et al. [11], ESR ≤ 92 mm/hour, neutrophils ≤ 78%, and WBC ≤ 9,700/mm^3^ are highly suggestive diagnostic markers for differentiating patients with TS, which is consistent with the blood tests of our two cases. A similar opinion was expressed by Kim et al., who noted that WBC count ≤ 10,000/mm^3^, neutrophil fraction up to 75%, ESR up to 40 mm/hour, CRP > 5 mg/dL, and alkaline phosphatase < 120 IU/L point to the diagnosis of TS [18].

Imaging diagnostics 

MRI is the diagnostic imaging method of choice for suspected spinal infection, with good sensitivity, specificity, and accuracy, especially after injection of a contrast agent [19]. The presence of a large, thin-walled paravertebral abscess, well-defined by the injected contrast agent, destruction of the endplates of the affected vertebrae and their destruction > 50%, and the presence of an intervertebral abscess are imaging findings that suggest the diagnosis of TS [11,20]. Contrast-enhanced MRI has been shown to show heterogeneous hyperintensity in 89.3% of cases [11].

The MRI findings in our two cases confirm the validity of the scoring system developed by Lertudomphonwanit et al., pointing towards the diagnosis of TS [11]. The total score of the scoring system ranges from 0 to 29 points, with the differentiation features including: thoracic location - 7 points; absence of epidural granulation tissue - 7 points; anterior subligamentous spread of infection - 5 points; intravertebral abscess - 3 points; well-defined paravertebral abscess - 2.5 points; presence of epidural abscess - 2.5 points, and absence of facet joint arthritis - 2 points. As the score increases, the level of concordance for making the diagnosis increases. In one of our patients, the sum was estimated at 20 points, and in the second, 22 points.

Surgical treatment

Surgical treatment in patients with TS is necessary in the presence of marked neurological deficit caused by spinal cord compression from an epidural abscess, sequestered bone and disc fragments, spinal deformity of the vertebral bodies > 50% with instability, severe or progressive kyphosis, and paraspinal abscesses [14]. The goal of surgical treatment is to ensure drainage of the abscess, removal of the infected tissue, debridement, and stabilization of the affected segments, thereby preventing progression of the deformity and promoting early neurological recovery in patients with neurological deficits [21]. Posterior instrumentation provides stability of the three columns of the affected vertebrae, sufficient exposure for spinal cord decompression, faster mobilization, and recovery of patients. The listed indications, the type, and the volume of the surgical interventions fully correspond to the reports in the literature. The success of the surgery depends on the optimal anti-tuberculosis treatment, according to the type of tuberculosis bacillus. The World Health Organization (WHO) recommends a nine-month treatment of spinal tuberculosis with four drugs given for two months (isoniazid, rifampicin, pyrazinamide, ethambutol or streptomycin), followed by isoniazid and rifampicin for seven months.

The risk of postoperative infection, tuberculosis-related and secondary pyogenic infections, is significant if tuberculosis is not completely controlled prior to surgery. The risk is increased because each revision increases scarring and hardware fatigue, and an active or simmering tuberculosis infection impairs local and systemic immunity. Before any revisional stabilization treatment, we must make sure the patient is on an effective anti-tuberculosis regimen with measurable clinical and microbiological improvement in order to avoid long-term stabilization failure and lower infection rates.

## Conclusions

The two cases of spinal tuberculosis presented illustrate the slow progression and nonspecific clinical symptoms of the disease, which hinder its timely diagnosis. This requires spinal tuberculosis to be included in the differential diagnosis of patients with vertebral pain and minimal neurological symptoms. In such patients, it is necessary to assess the leukocyte count, ESR, and CRP. If these values are elevated, even though they are not specific indicators, imaging studies should be performed: CT or, preferably, MRI, which offers the greatest diagnostic value. Visualization of a paravertebral abscess, pronounced destruction of the endplates of the affected vertebrae, and vertebral body collapse are imaging findings suggestive of spinal tuberculosis.

In the presence of significant neurological deficit, paravertebral and epidural abscesses, and more than 50% destruction of a vertebral body, urgent surgical decompression is indicated, including evacuation of the abscesses, debridement, and posterior instrumentation to stabilize the involved segment.
